# Type 2 Autoimmune Polyglandular Syndrome Presenting with Hyperpigmentation and Amenorrhea

**DOI:** 10.7759/cureus.7772

**Published:** 2020-04-21

**Authors:** Nayana Gaba, Saurabh Gaba, Mandeep Singla, Monica Gupta

**Affiliations:** 1 Obstetrics and Gynaecology, Postgraduate Institute of Medical Education and Research, Chandigarh, IND; 2 Internal Medicine, Government Medical College and Hospital, Chandigarh, IND; 3 General Medicine, Government Medical College and Hospital, Chandigarh, IND

**Keywords:** autoimmune polyglandular syndrome, schmidt syndrome, adrenal insufficiency, amenorrhea, aps

## Abstract

A 36-year-old female presented with lethargy, anorexia, nausea, hyperpigmentation, weight loss and amenorrhea for six months. On examination, she had hyperpigmentation of face, hands and oral mucosa. Investigations revealed adrenal insufficiency and subclinical hyperthyroidism with elevated anti-thyroid peroxidase antibodies. Adrenal insufficiency in combination with Grave’s disease and/or type 1 diabetes mellitus occurs in type 2 autoimmune polyglandular syndrome. It is a polygenic disorder occurring due to mutations in the human leukocyte antigen complex on chromosome 6. The patient was treated with oral hydrocortisone which led to improvement in all the symptoms.

## Introduction

Autoimmune polyglandular syndromes (APS) are a group of disorders that share a common genetic alteration that lead to dysfunction of multiple endocrine organs. These may be associated with a host of other autoimmune conditions. Autoimmunity can affect the adrenals, thyroid, pancreas, parathyroid glands, liver, gonads, skin and gastric mucosa [1]. The involvement of different organs can either be subtle and subclinical or it may be overt, leading to early diagnosis. Herein, the case of a middle-aged female with type 2 APS is presented. She had adrenal insufficiency, for which oral hydrocortisone was started, and subclinical Grave’s disease for which a wait and watch policy was adopted.

## Case presentation

**Figure 1:**
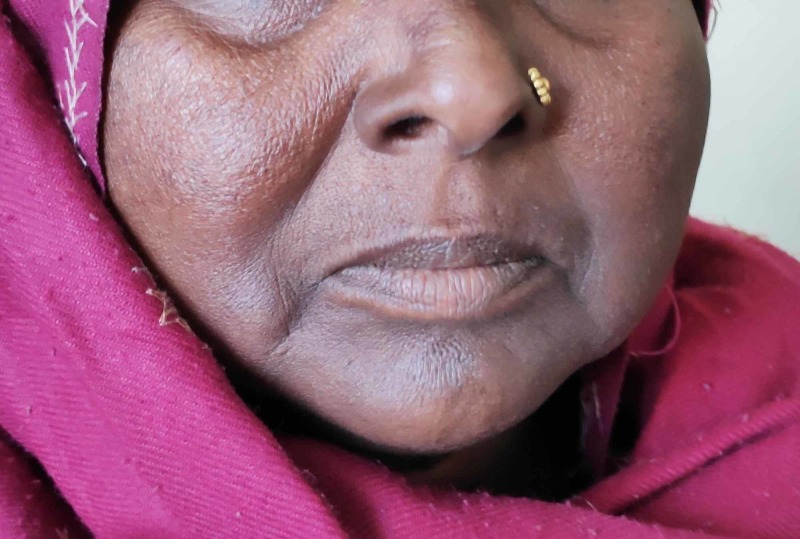
Hyperpigmentation of the face, more marked in the perioral region.

**Figure 2:**
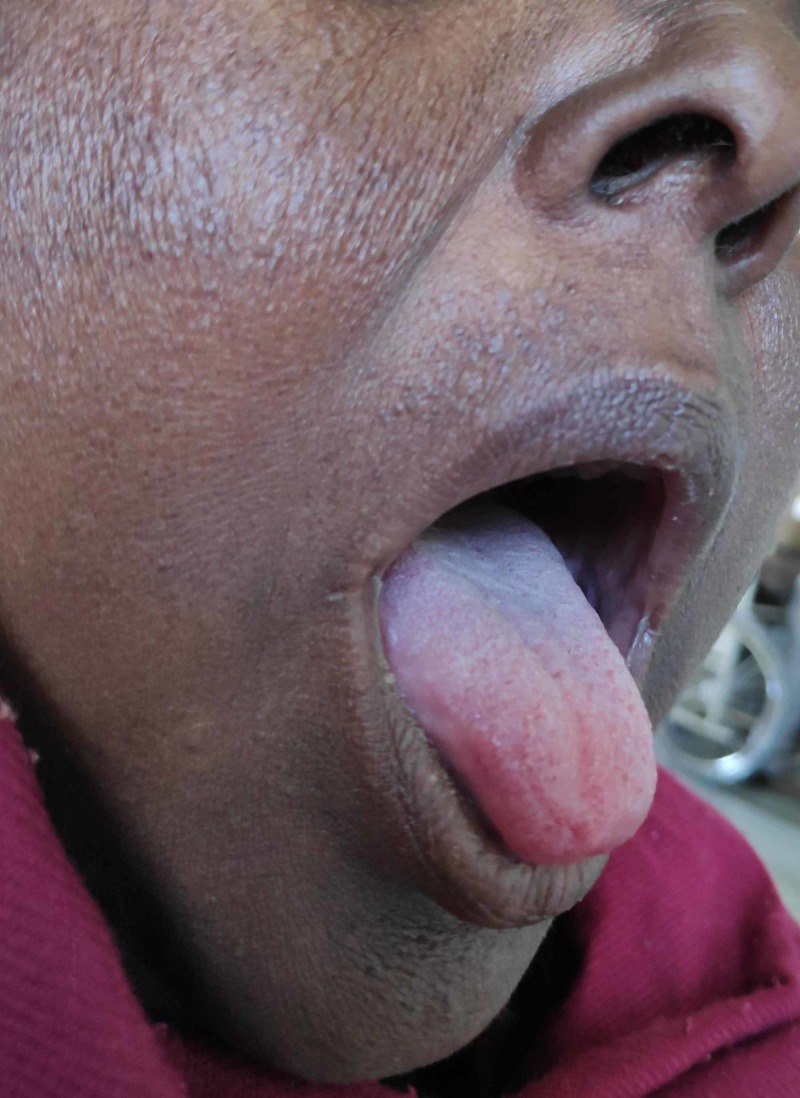
Hyperpigmentation of the tongue and buccal musoca.

**Figure 3:**
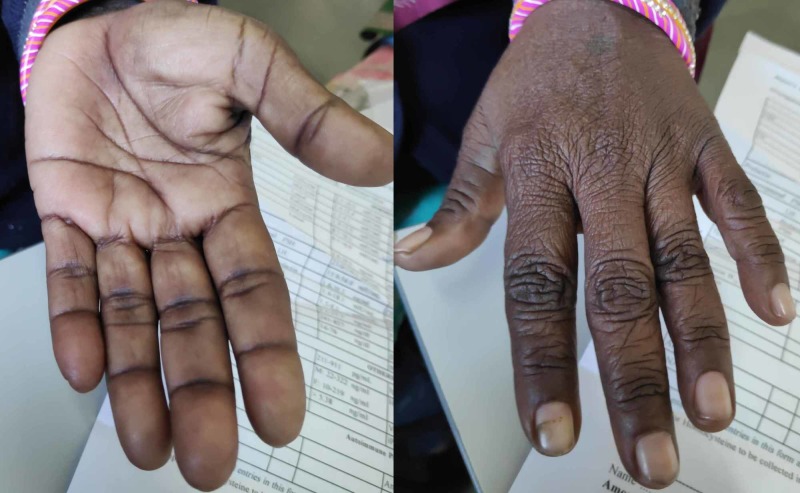
Hyperpigmentation of the palmar creases and dorsum of hands.

A 36-year-old female presented to the outpatient department with a history of anorexia, nausea, lethargy, undue fatigue, hyperpigmentation of skin and weight loss. The symptoms appeared over a period of months and worsened gradually to the point where she was unable to carry out her daily activities without assistance. She had amenorrhea for six months and occasional diarrhea without blood or nocturnal awakening. There was no history of chest pain, palpitations and shortness of breath. On examination, she was afebrile with a blood pressure of 108/70 mmHg and a regular pulse of 85 beats per minute. She had conspicuous hyperpigmentation of face, oral mucosa, dorsum of hand and palmar creases (Figures 1-3).

Respiratory, abdominal and cardiovascular examinations were unremarkable, and she did not have goiter or ophthalmopathy. Investigations revealed subclinical thyrotoxicosis with elevated anti-thyroid peroxidase antibody (anti-TPO) and low fasting cortisol with high concurrent adrenocorticotropic hormone (ACTH) (Table 1). Oral hydrocortisone replacement was started with thrice daily dosing: 7.5 mg in morning, 2.5 mg in afternoon and 2.5 mg in evening. This resulted in drastic symptomatic improvement. Menstural cycles also normalized with treatment, suggesting that amenorrhea was due to adrenal insufficiency per se, rather than hypogonadism. 

**Table 1:** Clinical investigations. AST, aspartate transaminase; ALT, alanine transaminase; ACTH, adrenocorticotropic hormone; TSH, thyroid-stimulating hormone; T3, triiodothyronine; T4, tetraiodothyronine; TPO, thyroid peroxidase; LH, luteinizing hormone; FSH, follicular-stimulating hormone; HbA1c, glycated hemoglobin. *Level depends on the phase of menstural cycle. Levels near the upper limit are seen in the perimenstural period.

Investigation	Value	Normal range
Sodium (mmol/L)	133	135-145
Potassium (mmol/L)	4.7	3.5-5.5
Urea (mg/dL)	32	15-40
Creatinine (mg/dL)	1.2	<1.3
Hemoglobin (g/dL)	12.9	13-15
Platelets (X10^9/L)	230	150-400
Total leukocyte count (X10^9/L)	6.4	4-12
AST (U/L)	37	10-40
ALT (U/L)	41	10-40
Morning cortisol (nmol/L)	67	200-600
Morning ACTH (pmol/L)	321	<80
TSH (mIU/L)	0.3	0.5-5
Free T3 (pg/dL)	440	260-480
Free T4 (ng/dL)	1.7	0.7-1.8
Anti-TPO (IU/mL)	>1000	<35
LH* (IU/L)	63	1-70
FSH* (IU/L)	7.2	2.5-10
Estradiol* (pg/mL)	76	30-400
Progesterone* (ng/mL)	2.4	0.5-20
Prolactin (ng/mL)	27	2-25
HbA1c	5.3%	<6%

## Discussion

Adrenal insufficiency

The most common cause of primary adrenal insufficiency or Addison’s disease in developed countries is autoimmune adrenalitis, while that in developing countries is tuberculosis [2]. The other causes include abrupt withdrawal of corticosteroid therapy, metastases, fungal infections, adrenal infarction and adrenoleukodystrophy. In patients with a reduced adrenal reserve, antifungal drugs such as fluconazole and ketoconazole, opiates such as morphine and tramadol, and anesthetic drug etomidate can also precipitate clinical adrenal insufficiency [3]. The clinical features of acute and chronic adrenal insufficiency are summarized in Table 2 [4]. The biochemical hallmarks are hyponatremia, hyperkalemia and less commonly, hypoglycemia and hypercalcemia. Hyperpigmentation is a virtually universal finding in adrenal insufficiency. It occurs due to excessive synthesis of melanin in response to increased production of melanocyte-stimulating hormone (MSH) [5]. MSH is formed by cleavage of proopiomelanocortin (POMC), a prohormone that is also a precursor of ACTH. Cortisol deficiency amplifies the production of POMC in the pituitary gland.

**Table 2:** Clinical features of adrenal insufficiency.

Chronic adrenal insufficiency	Acute adrenal insufficiency (adrenal crisis)
Anorexia, nausea, vomiting, abdominal pain	Hypotension and shock
Weight loss	Anorexia, nausea, vomiting, abdominal pain
Fatigue	Fever
Skin and mucosal hyperpigmentation	Confusion, delirium, coma
Hypotension	
Salt craving	
Mood disorders, psychosis	
Loss of libido	
Amenorrhea	

A high serum ACTH level in the setting of low cortisol suggests that the pituitary gland is functioning appropriately and rules out secondary adrenal insufficiency. The gold standard for diagnosis of adrenal insufficiency is ACTH stimulation test which displays an inadequate elevation of cortisol 30 minutes after an intravenous or intramuscular administration of a standard dose of ACTH [6].

Adrenal crisis is potentially fatal, and requires prompt administration of intravenous fluids and hydrocortisone bolus of 100 mg followed by 50 mg six hourly [7]. The preferred regime for chronic adrenal insufficiency is oral hydrocortisone taken in three divided doses, with around half of the total dose taken in morning to mimic the natural circadian rhythm of cortisol [8]. Total daily dose of 10-15 mg is generally required and the dose is titrated according to the symptoms. Fludrocortisone is usually not required as hydrocortisone itself possesses some intrinsic mineralocorticoid activity.

Grave's disease

Grave’s disease is the most common cause of thyrotoxicosis and is characterized by hyperthyroidism, a diffuse goiter and ophthalmopathy. Less common features are localized dermopathy (pretibial myxedema) and thyroid acropachy (clubbing). The ophthalmopathy can be serious enough to cause blindness by optic nerve compression and exposure keratitis. Excess glycosaminoglycan production by fibroblasts mediated by thyroid-stimulating hormone receptor stimulation in the extraocular tissues leads to proptosis and complex ophthalmoplegia [9]. A CT scan may sometimes be indicated to better visualize the retro ocular tissues and determine appropriate management. The ophthalmopathy follows its own independent clinical course, irrespective of the thyroid status [10]. Smoking and use of radioiodine therapy can exacerbate the eye disease [11].

Treatment of Grave’s disease involves control of thyrotoxicosis by thionamides (carbimazole, methimazole and propylthiouracil), radioiodine therapy or surgery [12]. Carbimazole and methimazole are preferred due to once a day dosing, more rapid achievement of euthyroid status and lesser hepatotoxicity. Propylthiouracil is preferred in thyroid storm and in early pregnancy. Early symptomatic relief from sympathetic symptoms is achieved by the use of propranolol, a non-selective beta-blocker. Eye disease requires use of topical lubricants, eye patch and dark glasses. Severe ophthalmopathy requires corticosteroids and surgical debulking. Anti-thyroid treatment is warranted in subclinical thyrotoxicosis only under circumstances such as symptomatic disease, presence of osteoporosis, atrial fibrillation or infertility and age more than 65 years [13].

Autoimmune polyglandular syndromes

Clustering of autoimmune phenomena within an individual and within a family is well known. This is best exemplified by autoimmune adrenalitis. In a case series, 47% of patients with adrenal insufficiency due to autoimmune adrenalitis were found to have another coexisting autoimmune disorder [14]. These included hypothyroidism, Grave’s disease, pernicious anemia, Sjögren’s syndrome, vitiligo, hypoparathyroidism, type 1 diabetes mellitus, coeliac disease and premature menopause. A comparison of APS 1 and 2 is shown in Table 3 [1]. Occurrence of hypoglycemia in a patient with previously well-controlled type 1 diabetes is highly suggestive of APS 2.

**Table 3:** Differences between APS 1 and 2. APS, autoimmune polyglandular syndrome; AIRE, autoimmune regulator; HLA, human leukocyte antigen.

	APS 1	APS 2 (Schmidt syndrome)
Diagnosis	Two of the following: mucocutaneous candidiasis, hypoparathroidism, adrenal insufficiency	Adrenal insufficiency with autoimmune thyroid disease (hypothyroidism or Grave’s disease) and/or type 1 diabetes mellitus
Other associations	Type 1 diabetes mellitus, hypothyroidism, hypogonadism, pernicious anemia, autoimmune hepatitis, alopecia, vitiligo, ectodermal dysplasia	Celiac disease, vitiligo, alopecia, myasthenia gravis, pernicious anemia, IgA deficiency, hypogonadism, autoimmune hepatitis
Cause	Mutations in the AIRE gene on chromosome 21	Mutations in DQ and DR regions of HLA complex on chromosome 6
Inheritance	Autosomal recessive	Polygenic
Peak prevalence age	5-10 years	20-40 years
Sex distribution	Male = Female	Female > Males
Incidence	<1 per 100,000/year	1-2 per 10,000/year

The treatment depends on the manifestations. Hormone replacement, anti-thyroid drugs and immunosuppression are used as indicated. It is essential is avoid treating hypothyroidism alone in the presence of adrenal insufficiency, as it can precipitate adrenal crisis due to enhanced corticosteroid metabolism in liver [15]. Antifungal drugs and supplementation with calcium and vitamin D are indicated in APS 1.

## Conclusions

This report describes a 36-year-old female with amenorrhea accompanied by obvious signs and symptoms of Addison’s disease, which was confirmed on investigations. The presence of subclinical Grave’s disease pointed toward a diagnosis of APS 2. Normal HbA1c and sex hormones ruled out concurrent diabetes and hypogonadism. Oral hydrocortisone was started with thrice a day dosing, and she experienced marked symptomatic improvement with correction of amenorrhea. APS 2 comprises of adrenal insufficiency with either or both of type 1 diabetes mellitus and autoimmune thyroid disease. Both types of APS are associated with a variety of other autoimmune disorders that need to be carefully sought and treated.
